# DNA fingerprinting in forensics: past, present, future

**DOI:** 10.1186/2041-2223-4-22

**Published:** 2013-11-18

**Authors:** Lutz Roewer

**Affiliations:** 1 Department of Forensic Genetics, Institute of Legal Medicine and Forensic Sciences, Charité - Universitätsmedizin Berlin, Berlin, Germany

**Keywords:** DNA fingerprinting, Forensic DNA profiling, Short tandem repeat, Lineage markers, Ancestry informative markers, Forensic DNA database, Privacy rights, Short tandem repeat

## Abstract

DNA fingerprinting, one of the great discoveries of the late 20th century, has revolutionized forensic investigations. This review briefly recapitulates 30 years of progress in forensic DNA analysis which helps to convict criminals, exonerate the wrongly accused, and identify victims of crime, disasters, and war. Current standard methods based on short tandem repeats (STRs) as well as lineage markers (Y chromosome, mitochondrial DNA) are covered and applications are illustrated by casework examples. Benefits and risks of expanding forensic DNA databases are discussed and we ask what the future holds for forensic DNA fingerprinting.

## The past - a new method that changed the forensic world

'“I’ve found it! I’ve found it”, he shouted, running towards us with a test-tube in his hand. “I have found a re-agent which is precipitated by hemoglobin, and by nothing else”,’ says Sherlock Holmes to Watson in Arthur Conan Doyle’s first novel *A study in Scarlet* from1886 and later: 'Now we have the Sherlock Holmes’ test, and there will no longer be any difficulty […]. Had this test been invented, there are hundreds of men now walking the earth who would long ago have paid the penalty of their crimes’ [1].

**Figure 1:**
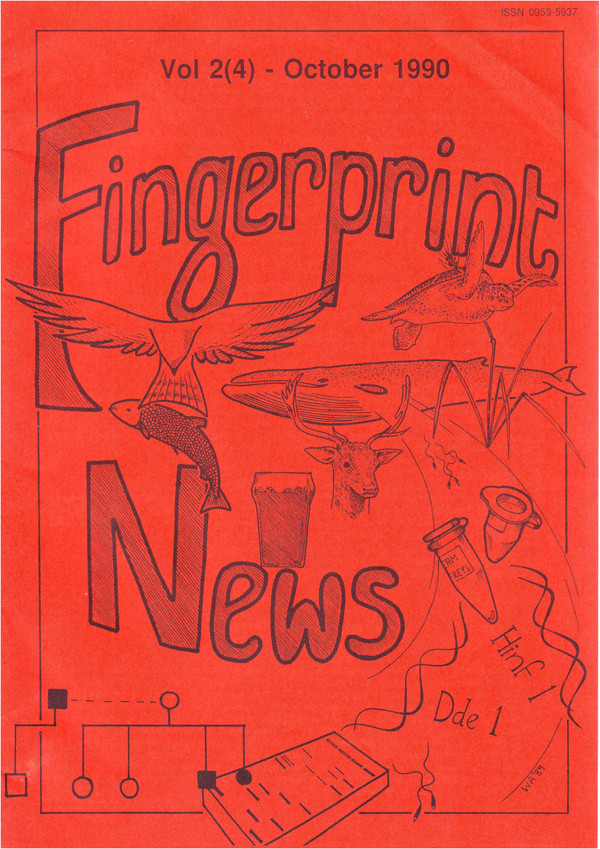
**Cover of one of the first issues of *Fingerprint News *** **from 1990.**

The Eureka shout shook England again and was heard around the world when roughly 100 years later Alec Jeffreys at the University of Leicester, in UK, found extraordinarily variable and heritable patterns from repetitive DNA analyzed with multi-locus probes. Not being Holmes he refrained to call the method after himself but 'DNA fingerprinting’ [2]. Under this name his invention opened up a new area of science. The technique proved applicable in many biological disciplines, namely in diversity and conservation studies among species, and in clinical and anthropological studies. But the true political and social dimension of genetic fingerprinting became apparent far beyond academic circles when the first applications in civil and criminal cases were published. Forensic genetic fingerprinting can be defined as the comparison of the DNA in a person’s nucleated cells with that identified in biological matter found at the scene of a crime or with the DNA of another person for the purpose of identification or exclusion. The application of these techniques introduces new factual evidence to criminal investigations and court cases. However, the first case (March 1985) was not strictly a forensic case but one of immigration [3]. The first application of DNA fingerprinting saved a young boy from deportation and the method thus captured the public’s sympathy. In Alec Jeffreys’ words: 'If our first case had been forensic I believe it would have been challenged and the process may well have been damaged in the courts’ [4]. The forensic implications of genetic fingerprinting were nevertheless obvious, and improvements of the laboratory process led already in 1987 to the very first application in a forensic case. Two teenage girls had been raped and murdered on different occasions in nearby English villages, one in 1983, and the other in 1986. Semen was obtained from each of the two crime scenes. The case was spectacular because it surprisingly excluded a suspected man, Richard Buckland, and matched another man, Colin Pitchfork, who attempted to evade the DNA dragnet by persuading a friend to give a sample on his behalf. Pitchfork confessed to committing the crimes after he was confronted with the evidence that his DNA profile matched the trace DNA from the two crime scenes. For 2 years the Lister Institute of Leicester where Jeffreys was employed was the only laboratory in the world doing this work. But it was around 1987 when companies such as Cellmark, the academic medico-legal institutions around the world, the national police, law enforcement agencies, and so on started to evaluate, improve upon, and employ the new tool. The years after the discovery of DNA fingerprinting were characterized by a mood of cooperation and interdisciplinary research. None of the many young researchers who has been there will ever forget the DNA fingerprint congresses which were held on five continents, in Bern (1990), in Belo Horizonte (1992), in Hyderabad (1994), in Melbourne (1996), and in Pt. Elizabeth (1999), and then shut down with the good feeling that the job was done. Everyone read the *Fingerprint News* distributed for free by the University of Cambridge since 1989 (Figure 1). This affectionate little periodical published non-stylish short articles directly from the bench without impact factors and resumed networking activities in the different fields of applications. The period in the 1990s was the golden research age of DNA fingerprinting succeeded by two decades of engineering, implementation, and high-throughput application. From the Foreword of Alec Jeffreys in *Fingerprint News*, Issue 1, January 1989: 'Dear Colleagues, […] I hope that Fingerprint News will cover all aspects of hypervariable DNA and its application, including both multi-locus and single-locus systems, new methods for studying DNA polymorphisms, the population genetics of variable loci and the statistical analysis of fingerprint data, as well as providing useful technical tips for getting good DNA profiles […]. May your bands be variable’ [5].

Jeffreys’ original technology, now obsolete for forensic use, underwent important developments in terms of the basic methodology, that is, from Southern blot to PCR, from radioactive to fluorescent labels, from slab gels to capillary electrophoresis. As the technique became more sensitive, the handling simple and automated and the statistical treatment straightforward, DNA profiling, as the method was renamed, entered the forensic routine laboratories around the world in storm. But, what counts in the Pitchfork case and what still counts today is the process to get DNA identification results accepted in legal proceedings. Spectacular fallacies, from the historical 1989 case of People *vs.* Castro in New York [6] to the case against Knox and Sollecito in Italy (2007–2013) where literally DNA fingerprinting was on trial [7], disclosed severe insufficiencies in the technical protocols and especially in the DNA evidence interpretation and raised *nolens volens* doubts on the scientific and evidentiary value of forensic DNA fingerprinting. These cases are rare but frequent enough to remind each new generation of forensic analysts, researchers, or private sector employees that DNA evidence is nowadays an important part of factual evidence and needs thus intense scrutiny for all parts of the DNA analysis and interpretation process.

In the following I will briefly describe the development of DNA fingerprinting to a standardized investigative method for court use which has since 1984 led to the conviction of thousands of criminals and to the exoneration of many wrongfully suspected or convicted individuals [8]. Genetic fingerprinting *per se* could of course not reduce the criminal rate in any of the many countries in the world, which employ this method. But DNA profiling adds hard scientific value to the evidence and strengthens thus (principally) the credibility of the legal system.

## The technological evolution of forensic DNA profiling

**Figure 2:**
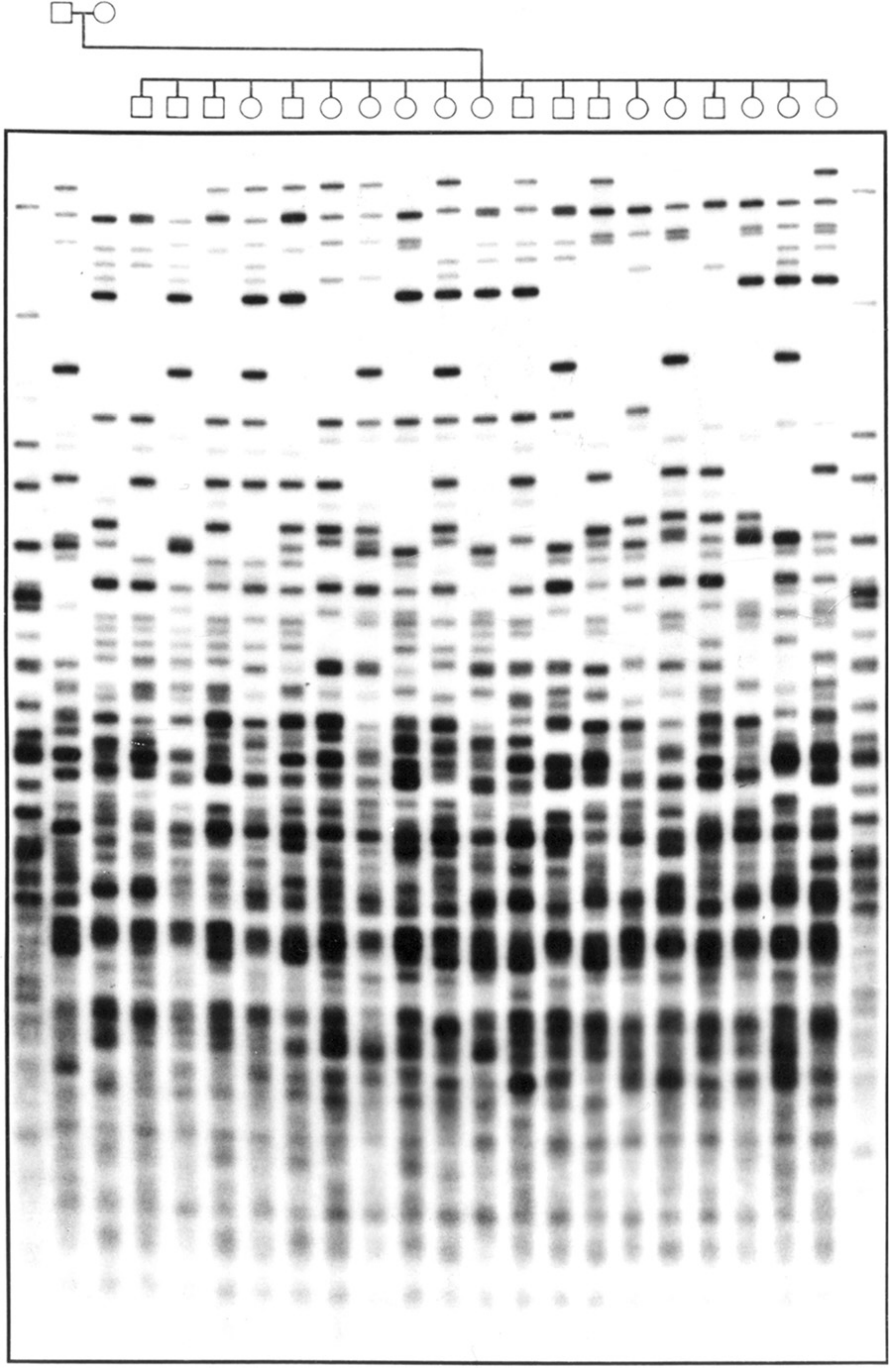
**Multilocus DNA Fingerprint from a large family probed with the oligonucleotide (GTG)**_**5 **_**(*Courtesy of Peter Nürnberg, Cologne Center for Genomics, Germany*).**

In the classical DNA fingerprinting method radio-labeled DNA probes containing minisatellite [9] or oligonucleotide sequences [10] are hybridized to DNA that has been digested with a restriction enzyme, separated by agarose electrophoresis and immobilized on a membrane by Southern blotting or - in the case of the oligonucleotide probes - immobilized directly in the dried gel. The radio-labeled probe hybridizes to a set of minisatellites or oligonucleotide stretches in genomic DNA contained in restriction fragments whose size differ because of variation in the numbers of repeat units. After washing away excess probe the exposure to X-ray film (autoradiography) allows these variable fragments to be visualized, and their profiles compared between individuals. Minisatellite probes, called 33.6 and 33.15, were most widely used in the UK, most parts of Europe and the USA, whereas pentameric (CAC)/(GTG)_5_ probes were predominantly applied in Germany. These so-called multilocus probes (MLP) detect sets of 15 to 20 variable fragments per individual ranging from 3.5 to 20 kb in size (Figure 2). But the multi-locus profiling method had several limitations despite its successful application to crime and kinship cases until the middle of the 1990s. Running conditions or DNA quality issues render the exact matching between bands often difficult. To overcome this, forensic laboratories adhered to binning approaches [11], where fixed or floating bins were defined relative to the observed DNA fragment size, and adjusted to the resolving power of the detection system. Second, fragment association within one DNA fingerprint profile is not known, leading to statistical errors due to possible linkage between loci. Third, for obtaining optimal profiles the method required substantial amounts of high molecular weight DNA [12] and thus excludes the majority of crime-scene samples from the analysis. To overcome some of these limitations, single-locus profiling was developed [13]. Here a single hypervariable locus is detected by a specific single-locus probe (SLP) using high stringency hybridization. Typically, four SLPs were used in a reprobing approach, yielding eight alleles of four independent loci per individual. This method requires only 10 ng of genomic DNA [14] and has been validated through extensive experiments and forensic casework, and for many years provided a robust and valuable system for individual identification. Nevertheless, all these different restriction fragment length polymorphism (RFLP)-based methods were still limited by the available quality and quantity of the DNA and also hampered by difficulties to reliably compare genetic profiles from different sources, labs, and techniques. What was needed was a DNA code, which could ideally be generated even from a single nucleated cell and from highly degraded DNA, a code, which could be rapidly generated, numerically encrypted, automatically compared, and easily supported in court. Indeed, starting in the early 1990s DNA fingerprinting methods based on RFLP analysis were gradually supplanted by methods based on PCR because of the improved sensitivity, speed, and genotyping precision [15]. Microsatellites, in the forensic community usually referred to short tandem repeats (STRs), were found to be ideally suited for forensic applications. STR typing is more sensitive than single-locus RFLP methods, less prone to allelic dropout than VNTR (variable number of tandem repeat) systems [16], and more discriminating than other PCR-based typing methods, such as HLA-DQA1 [17]. More than 2,000 publications now detail the technology, hundreds of different population groups have been studied, new technologies as, for example, the miniSTRs [18] have been developed and standard protocols have been validated in laboratories worldwide (for an overview see [19]). Forensic DNA profiling is currently performed using a panel of multi-allelic STR markers which are structurally analogous to the original minisatellites but with much shorter repeat tracts and thus easier to amplify and multiplex with PCR. Up to 30 STRs can be detected in a single capillary electrophoresis injection generating for each individual a unique genetic code. Basically there are two sets of STR markers complying with the standards requested by criminal databases around the world: the European standard set of 12 STR markers [20] and the US CODIS standard of 13 markers [21]. Due to partial overlap, they form together a standard of 18 STR markers in total. The incorporation of these STR markers into commercial kits has improved the application of these markers for all kinds of DNA evidence with reproducible results from as less than three nucleated cells [22] and extracted even from severely compromised material. The probability that two individuals will have identical markers at each of 13 different STR loci within their DNA exceeds one out of a billion. If a DNA match occurs between an accused individual and a crime scene stain, the correct courtroom expression would be that the probability of a match if the crime-scene sample came from someone other than the suspect (considering the random, not closely-related man) is at most one in a billion [14]. The uniqueness of each person’s DNA (with the exception of monozygotic twins) and its simple numerical codification led to the establishment of government-controlled criminal investigation DNA databases in the developed nations around the world, the first in 1995 in the UK [23]. When a match is made from such a DNA database to link a crime scene sample to an offender who has provided a DNA sample to a database that link is often referred to as a cold hit. A cold hit is of value as an investigative lead for the police agency to a specific suspect. China (approximately 16 million profiles, the United States (approximately 10 million profiles), and the UK (approximately 6 million profiles) maintain the largest DNA database in the world. The percentage of databased persons is on the increase in all countries with a national DNA database, but the proportions are not the same by the far: whereas in the UK about 10% of the population is in the national DNA database, the percentage in Germany and the Netherlands is only about 0.9% and 0.8%, respectively [24].

## Lineage markers in forensic analysis

**Figure 3:**
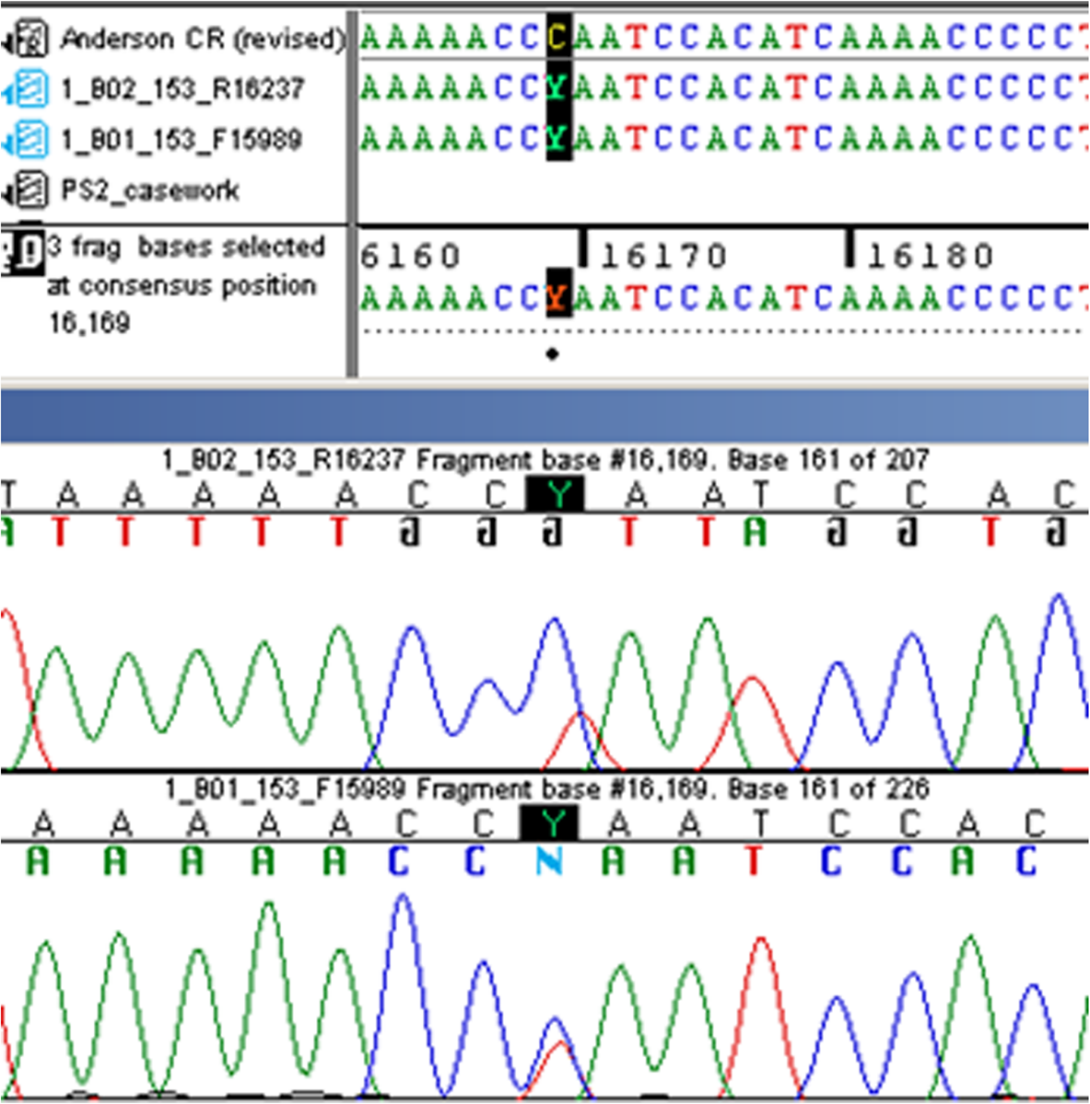
**Screenshot of the 16169 C/T heteroplasmy present in Tsar Nicholas II using both forward and reverse sequencing primers (*Courtesy of Michael Coble, National Institute of Standards and Technology, Gaithersburg, USA*).**

**Figure 4:**
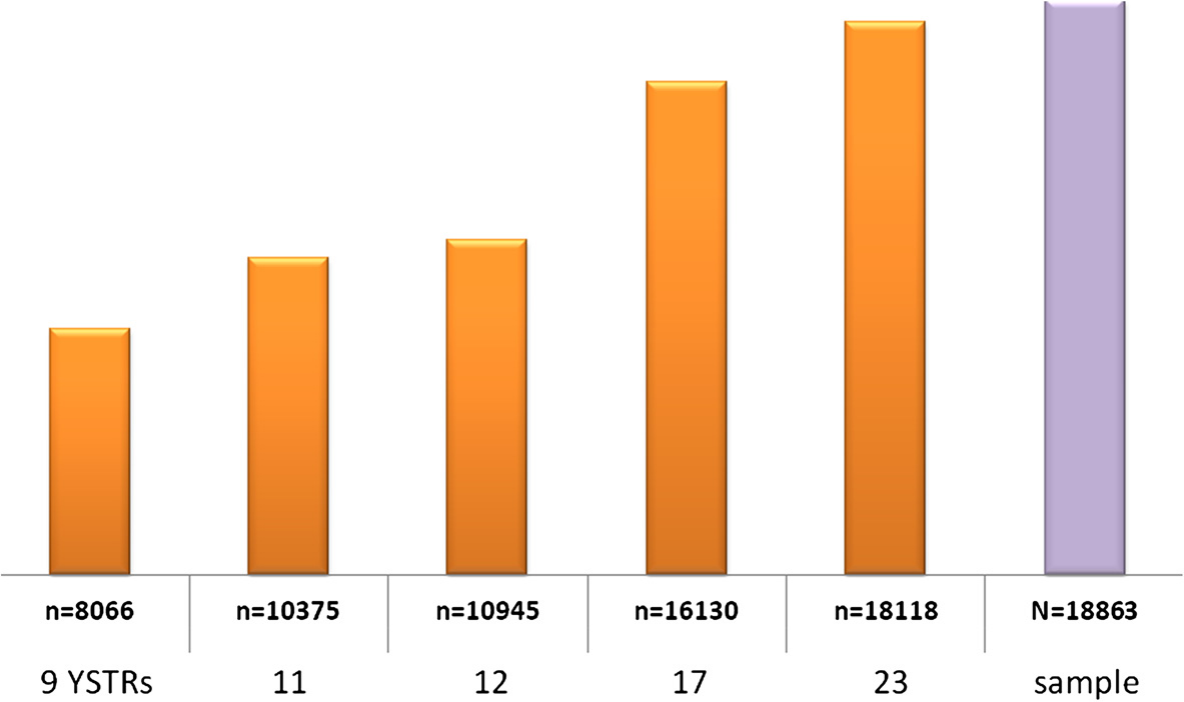
Correlation between the number of analyzed Y-STRs and the number of different haplotypes detected in a global population sample of 18,863 23-locus haplotypes.

Lineage markers have special applications in forensic genetics. Y chromosome analysis is very helpful in cases where there is an excess of DNA from a female victim and only a low proportion from a male perpetrator. Typical examples include sexual assault without ejaculation, sexual assault by a vasectomized male, male DNA under the fingernails of a victim, male 'touch’ DNA on the skin, and the clothing or belongings of a female victim. Mitochondrial DNA (mtDNA) is of importance for the analyses of low level nuclear DNA samples, namely from unidentified (typically skeletonized) remains, hair shafts without roots, or very old specimens where only heavily degraded DNA is available [25]. The unusual non-recombinant mode of inheritance of Y and mtDNA weakens the statistical weight of a match between individual samples but makes the method efficient for the reconstruction of the paternal or maternal relationship, for example in mass disaster investigations [26] or in historical reconstructions. A classic case is the identification of two missing children of the Romanov family, the last Russian monarchy. MtDNA analysis combined with additional DNA testing of material from the mass grave near Yekaterinburg gave virtually irrefutable evidence that the two individuals recovered from a second grave nearby are the two missing children of the Romanov family: the Tsarevich Alexei and one of his sisters [27]. Interestingly, a point heteroplasmy, that is, the presence of two slightly different mtDNA haplotypes within an individual, was found in the mtDNA of the Tsar and his relatives, which was in 1991 a contentious finding (Figure 3). In the early 1990s when the bones were first analyzed, a point heteroplasmy was believed to be an extremely rare phenomenon and was not readily explainable. Today, the existence of heteroplasmy is understood to be relatively common and large population databases can be searched for its frequency at certain positions. The mtDNA evidence in the Romanov case was underpinned by Y-STR analysis where a 17-locus haplotype from the remains of Tsar Nicholas II matched exactly to the femur of the putative Tsarevich and also to a living Romanov relative. Other studies demonstrated that very distant family branches can be traced back to common ancestors who lived hundreds of years ago [28]. Currently forensic Y chromosome typing has gained wide acceptance with the introduction of highly sensitive panels of up to 27 STRs including rapidly mutating markers [29]. Figure 4 demonstrates the impressive gain of the discriminative power with increasing numbers of Y-STRs. The determination of the match probability between Y-STR or mtDNA profiles via the mostly applied counting method [30] requires large, representative, and quality-assessed databases of haplotypes sampled in appropriate reference populations, because the multiplication of individual allele frequencies is not valid as for independently inherited autosomal STRs [31]. Other estimators for the haplotype match probability than the count estimator have been proposed and evaluated using empirical data [32], however, the biostatistical interpretation remains complicated and controversial and research continues. The largest forensic Y chromosome haplotype database is the YHRD (http://www.yhrd.org) hosted at the Institute of Legal Medicine and Forensic Sciences in Berlin, Germany, with about 115,000 haplotypes sampled in 850 populations [33]. The largest forensic mtDNA database is EMPOP (http://www.empop.org) hosted at the Institute of Legal Medicine in Innsbruck, Austria, with about 33,000 haplotypes sampled in 63 countries [34]. More than 235 institutes have actually submitted data to the YHRD and 105 to EMPOP, a compelling demonstration of the level of networking activities between forensic science institutes around the world. That additional intelligence information is potentially derivable from such large datasets becomes obvious when a target DNA profile is searched against a collection of geographically annotated Y chromosomal or mtDNA profiles. Because linearly inherited markers have a highly non-random geographical distribution the target profile shares characteristic variants with geographical neighbors due to common ancestry [35]. This link between genetics, genealogy, and geography could provide investigative leads for investigators in non-suspect cases as illustrated in the following case [36]:

**Figure 5:**
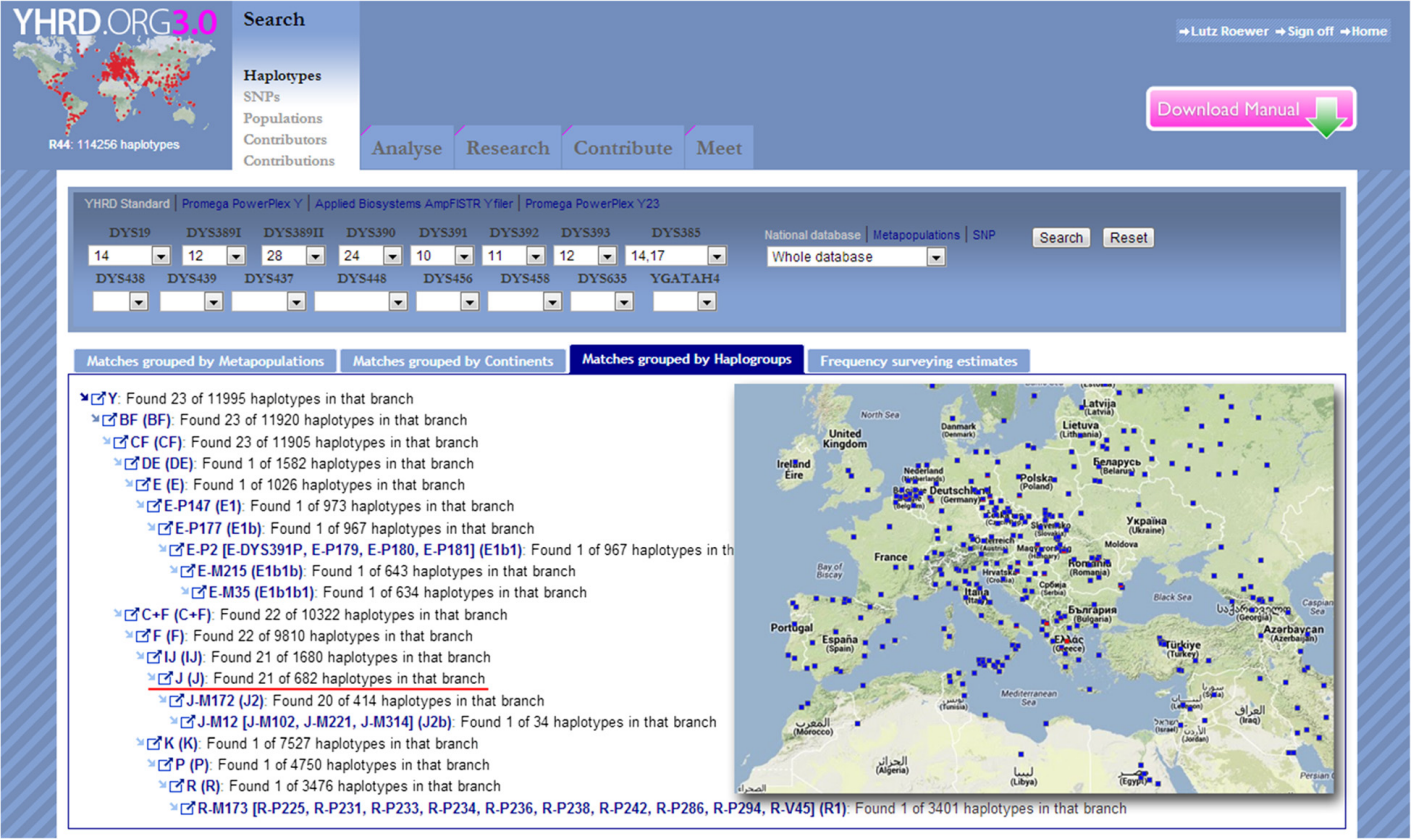
Screenshot from the YHRD depicting the radiation of a 9-locus haplotype belonging to haplogroup J in Southern Europe.

In 2002, a woman was found with a smashed skull and covered in blood but still alive in her Berlin apartment. Her life was saved by intensive medical care. Later she told the police that she had let a man into her apartment, and he had immediately attacked her. The man was subletting the apartment next door. The evidence collected at the scene and in the neighboring apartment included a baseball cap, two towels, and a glass. The evidence was sent to the state police laboratory in Berlin, Germany and was analyzed with conventional autosomal STR profiling. Stains on the baseball cap and on one towel revealed a pattern consistent with that of the tenant, whereas two different male DNA profiles were found on a second bath towel and on the glass. The tenant was eliminated as a suspect because he was absent at the time of the offense, but two unknown men (different in autosomal but identical in Y-STRs) who shared the apartment were suspected. Unfortunately, the apartment had been used by many individuals of both European and African nationalities, so the initial search for the two men became very difficult. The police obtained a court order for Y-STR haplotyping to gain information about the unknown men’s population affiliation. Prerequisites for such biogeographic analyses are large reference databases containing Y-STR haplotypes also typed for ancestry informative single nucleotide markers (SNP) markers from hundreds of different populations. The YHRD proved useful to infer the population origin of the unknown man. The database inquiry indicated a patrilineage of Southern European ancestry, whereas an African descent was unlikely (Figure 5). The police were able to track down the tenant in Italy, and with his help, establish the identity of one of the unknown men, who was also Italian. When questioning this man, the police used the information retrieved from Y-STR profiling that he had shared the apartment in Berlin with a paternal relative. This relative was identified as his nephew. Because of the close-knit relationship within the family, this information would probably not have been easily retrieved from the uncle without the prior knowledge. The nephew was suspected of the attempted murder in Berlin. He was later arrested in Italy, where he had committed another violent robbery.

Information on the biogeographic origin of an unknown DNA could also be retrieved from a number of ancestry informative SNPs (AISNPs) on autosomes or insertion/deletion polymorphisms [37,38] but perhaps even better from so-called mini-haplotypes with only <10 SNPs spanning small molecular intervals (<10 kb) with very low recombination among sites [39]. Each 'minihap’ behaves like a locus with multiple haplotype lineages (alleles) that have evolved from the ancestral human haplotype. All copies of each distinct haplotype are essentially identical by descent. Thus, they fall like Y and mtDNA into the lineage-informative category of genetic markers and are thus useful for connecting an individual to a family or ancestral genetic pool.

## Benefits and risks of forensic DNA databases

The steady growth in the size of forensic DNA databases raises issues on the criteria of inclusion and retention and doubts on the efficiency, commensurability, and infringement of privacy of such large personal data collections. In contrast to the past, not only serious but all crimes are subject to DNA analysis generating millions and millions of DNA profiles, many of which are stored and continuously searched in national DNA databases. And as always when big datasets are gathered new mining procedures based on correlation became feasible. For example, 'Familial DNA Database Searching’ is based on near matches between a crime stain and a databased person, which could be a near relative of the true perpetrator [40]. Again the first successful familial search was conducted in UK in 2004 and led to the conviction of Craig Harman of manslaughter. Craig Harman was convicted because of partial matches from Harman’s brother. The strategy was subsequently applied in some US states but is not conducted at the national level. It was during a dragnet that it first became public knowledge that the German police were also already involved in familial search strategies. In a little town in Northern Germany the police arrested a young man accused of rape because they had analyzed the DNA of his two brothers who had participated in the dragnet. Because of partial matches between crime scene DNA profiles and these brothers they had identified the suspect. In contrast to other countries, the Federal Constitutional Court of Germany decided in December 2012 against the future court use of this kind of evidence.

Civil rights and liberties are crucial for democratic societies and plans to extend forensic DNA databases to whole populations need to be condemned. Alec Jeffreys early on has questioned the way UK police collects DNA profiles, holding not only convicted individuals but also arrestees without conviction, suspects cleared in an investigation, or even innocent people never charged with an offence [41]. He also criticized that large national databases as the NDNAD of England and Wales are likely skewed socioeconomically. It has been pointed out that most of the matches refer to minor offences; according to *GeneWatch* in Germany 63% of the database matches provided are related to theft while <3% related to rape and murder. The changes to the UK database came in the 2012’s Protection of Freedoms bill, following a major defeat at the European Court of Human Rights in 2008. As of May 2013 1.1 million profiles (of about 7 million) had been destroyed to remove innocent people’s profiles from the database. In 2005 the incoming government of Portugal proposed a DNA database containing samples from every Portuguese citizen. Following public objections, the government limited the database to criminals. A recent study on the public views on DNA database-related matters showed that a more critical attitude towards wider national databases is correlated with the age and education of the respondents [42]. A deeper public awareness on the benefits and risks of very large DNA collections need to be built and common ethical and privacy standards for the development and governance of DNA databases need to be adopted where the citizen’s perspectives are taken into consideration.

## The future of forensic DNA analysis

**Figure 6:**
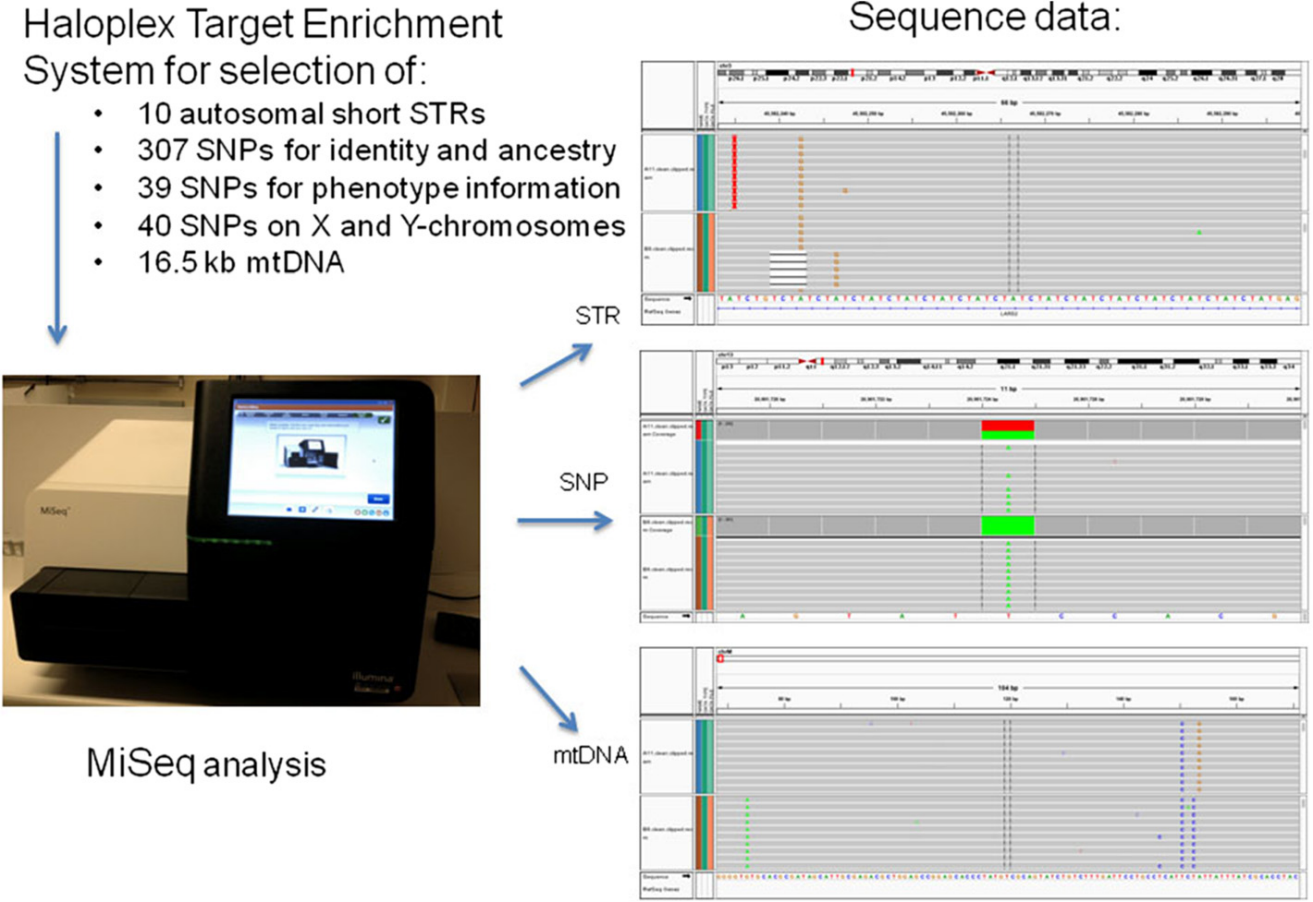
**Schematic overview of Haloplex targeting and NGS analysis of a large number of markers simultaneously.** Sequence data are shown for samples from two individuals and the D3S1358 STR marker, the rs1335873 SNP marker, and a part of the HVII region of mtDNA (*Courtesy of Marie Allen, Uppsala University, Sweden*).

The forensic community, as it always has, is facing the question in which direction the DNA Fingerprint technology will be developed. A growing number of colleagues are convinced that DNA sequencing will soon replace methods based on fragment length analysis and there are good arguments for this position. With the emergence of current Next Generation Sequencing (NGS) technologies, the body of forensically useful data can potentially be expanded and analyzed quickly and cost-efficiently. Given the enormous number of potentially informative DNA loci - which of those should be sequenced? In my opinion there are four types of polymorphisms which deserve a place on the analytic device: an array of 20–30 autosomal STRs which complies with the standard sets used in the national and international databases around the world, a highly discriminating set of Y chromosomal markers, individual and signature polymorphisms in the control and coding region of the mitochondrial genome [43], as well as ancestry and phenotype inference SNPs [44]. Indeed, a promising NGS approach with the simultaneous analysis of 10 STRs, 386 autosomal ancestry and phenotype informative SNPs, and the complete mtDNA genome has been presented recently [45] (Figure 6). Currently, the rather high error rates are preventing NGS technologies from being used in forensic routine [46], but it is foreseeable that the technology will be improved in terms of accuracy and reliability. Time is another essential factor in police investigations which will be considerably reduced in future applications of DNA profiling. Commercial instruments capable of producing a database-compatible DNA profile within 2 hours exist [47] and are currently under validation for law enforcement use. The hands-free 'swab in - profile out’ process consists of automated extraction, amplification, separation, detection, and allele calling without human intervention. In the US the promise of on-site DNA analysis has already altered the way in which DNA could be collected in future. In a recent decision the Supreme court of the United States held that 'when officers make an arrest supported by probable cause to hold for a serious offense and bring the suspect to the station to be detained in custody, taking and analyzing a cheek swab of the arrestee’s DNA is, like fingerprinting and photographing, a legitimate police booking procedure’ (Maryland *v.* Alonzo Jay King, Jr.). In other words, DNA can be taken from any arrestee, rightly or wrongly arrested, as a part of the normal booking procedure. Twenty-eight states and the federal government now take DNA swabs after arrests with the aim of comparing profiles to the CODIS database, creating links to unsolved cases and to identify the person (Associated Press, 3 June 2013). Driven by the rapid technological progress DNA actually becomes another metric of quick identification. It remains to be seen whether rapid DNA technologies will alter the way in which DNA is collected by police in other countries. In Germany for example the DNA collection is still regulated by the code of the criminal procedure and the use of DNA profiling for identification purposes only is excluded. Because national legislations are basically so different, a worldwide system to interrogate DNA profiles from criminal justice databases seems currently a very distant project.

At present the forensic DNA technology directly affects the lives of millions people worldwide. The general acceptance of this technique is still high, reports on the DNA identification of victims of the 9/11 terrorist attacks [48], of natural disasters as the Hurricane Katrina [49], and of recent wars (for example, in former Yugoslavia [50]) and dictatorship (for example, in Argentina [51]) impress the public in the same way as police investigators in white suits securing DNA evidence at a broken door. CSI watchers know, and even professionals believe, that DNA will inevitably solve the case just following the motto *Do Not Ask, it’s DNA, stupid!* But the affirmative view changes and critical questions are raised. It should not be assumed that the benefits of forensic DNA fingerprinting will necessarily override the social and ethical costs [52].

This short article leaves many of such questions unanswered. Alfred Nobel used his fortune to institute a prize for work 'in ideal direction’. What would be the ideal direction in which DNA fingerprinting, one of the great discoveries in recent history, should be developed?

## Competing interests

The author declares that he has no competing interests.

## References

[B1] Doyle AC A study in scarlet, Beeton’s Christmas Annual 1887 London, New York and Melbourne: Ward, Lock & Co

[B2] Jeffreys AJ Wilson V Thein SL Individual-specific “fingerprints” of Human DNA Nature 1985 314 67 74 10.1038/314067a0 2989708

[B3] Jeffreys AJ Brookfield JF Semeonoff R Positive identification of an immigration test-case using human DNA fingerprints Nature 1985 317 818 819 10.1038/317818a0 4058586

[B4] University of Leicester Bulletin Supplement August/September 2004

[B5] Jeffreys AJ Foreword Fingerprint News 1989 1 1

[B6] Lander ES DNA fingerprinting on trial Nature 1989 339 501 505 10.1038/339501a0 2567496

[B7] Balding DJ Evaluation of mixed-source, low-template DNA profiles in forensic science Proc Natl Acad Sci U S A 2013 110 12241 12246 10.1073/pnas.1219739110 23818643 PMC3725068

[B8] The innocence project[http://www.innocenceproject.org]

[B9] Jeffreys AJ Wilson V Thein SL Hypervariable 'minisatellite’ regions in human DNA Nature 1985 314 67 73 10.1038/314067a0 3856104

[B10] Schäfer R Zischler H Birsner U Becker A Epplen JT Optimized oligonucleotide probes for DNA fingerprinting Electrophoresis 1988 9 369 374 10.1002/elps.1150090804 3234377

[B11] Budowle B Giusti AM Waye JS Baechtel FS Fourney RM Adams DE Presley LA Deadman HA Monson KL Fixed-bin analysis for statistical evaluation of continuous distributions of allelic data from VNTR loci, for use in forensic comparisons Am J Hum Genet 1991 48 841 855 1673286 PMC1683046

[B12] Roewer L Nürnberg P Fuhrmann E Rose M Prokop O Epplen JT Stain analysis using oligonucleotide probes specific for simple repetitive DNA sequences Forensic Sci Int 1990 47 59 70 10.1016/0379-0738(90)90285-7 2210552

[B13] Wong Z Wilson V Patel I Povey S Jeffreys AJ Characterization of a panel of highly variable minisatellites cloned from human DNA Ann Hum Genet 1987 51 269 288 10.1111/j.1469-1809.1987.tb01062.x 3482146

[B14] Jobling MA Hurles ME Tyler-Smith C Chapter 15: Identity and identification Human Evolutionary Genetics 2003 Abingdon: Garland Science 474 497

[B15] Edwards A Civitello A Hammond HA Caskey CT DNA typing and genetic mapping with trimeric and tetrameric tandem repeats Am J Hum Genet 1991 49 746 756 1897522 PMC1683171

[B16] Budowle B Chakraborty R Giusti AM Eisenberg AJ Allen RC Analysis of the VNTR locus D1S80 by the PCR followed by high-resolution PAGE Am J Hum Genet 1991 48 137 144 1670750 PMC1682756

[B17] Saiki RK Bugawan TL Horn GT Mullis KB Erlich HA Analysis of enzymatically amplified beta-globin and HLA-DQ alpha DNA with allele-specific oligonucleotide probes Nature 1986 324 163 166 10.1038/324163a0 3785382

[B18] Coble MD Butler JM Characterization of new miniSTR loci to aid analysis of degraded DNA J Forensic Sci 2005 50 43 53 15830996

[B19] Butler JM Forensic DNA Typing: Biology, Technology, and Genetics of STR Markers 2005 2 New York: Elsevier Academic Press

[B20] Gill P Fereday L Morling N Schneider PM The evolution of DNA databases - Recommendations for new European STR loci Forensic Sci Int 2006 156 242 244 10.1016/j.forsciint.2005.05.036 16002250

[B21] Budowle B Moretti TR Niezgoda SJ Brown BL CODIS and PCR-based short tandem repeat loci: law enforcement tools Proceedings of the Second European Symposium on Human Identification 1998 Madison, WI: Promega Corporation 73 88

[B22] Nagy M Otremba P Krüger C Bergner-Greiner S Anders P Henske B Prinz M Roewer L Optimization and validation of a fully automated silica-coated magnetic beads purification technology in forensics Forensic Sci Int 2005 152 13 22 10.1016/j.forsciint.2005.02.027 15871915

[B23] Martin PD Schmitter H Schneider PM A brief history of the formation of DNA databases in forensic science within Europe Forensic Sci Int 2001 119 225 231 10.1016/S0379-0738(00)00436-9 11376988

[B24] ENFSI survey on DNA Databases in Europe. December 2011, published 2012-08-18 [http://www.enfsi.eu]

[B25] Roewer L Parson W Siegel JA, Saukko PJ Internet accessible population databases: YHRD and EMPOP Encyclopedia of Forensic Sciences 2013 2 Amsterdam: Elsevier B.V

[B26] Calacal GC Delfin FC Tan MM Roewer L Magtanong DL Lara MC Rd F De Ungria MC Identification of exhumed remains of fire tragedy victims using conventional methods and autosomal/Y-chromosomal short tandem repeat DNA profiling Am J Forensic Med Pathol 2005 26 285 291 10.1097/01.paf.0000177338.21951.82 16121088

[B27] Coble MD Loreille OM Wadhams MJ Edson SM Maynard K Meyer CE Niederstätter H Berger C Berger B Falsetti AB Gill P Parson W Finelli LN Mystery solved: the identification of the two missing Romanov children using DNA analysis PLoS One 2009 4 e4838 10.1371/journal.pone.0004838 19277206 PMC2652717

[B28] Haas C Shved N Rühli FJ Papageorgopoulou C Purps J Geppert M Willuweit S Roewer L Krawczak M Y-chromosomal analysis identifies the skeletal remains of Swiss national hero Jörg Jenatsch (1596–1639) Forensic Sci Int Genet 2013 7 610 617 10.1016/j.fsigen.2013.08.006 24035510

[B29] Ballantyne KN Keerl V Wollstein A Choi Y Zuniga SB Ralf A Vermeulen M de Knijff P Kayser M A new future of forensic Y-chromosome analysis: rapidly mutating Y-STRs for differentiating male relatives and paternal lineages Forensic Sci Int Genet 2012 6 208 218 10.1016/j.fsigen.2011.04.017 21612995

[B30] Budowle B Sinha SK Lee HS Chakraborty R Utility of Y-chromosome short tandem repeat haplotypes in forensic applications Forensic Sci Rev 2003 15 153 164 26256730

[B31] Roewer L Kayser M de Knijff P Anslinger K Betz A Caglià A Corach D Füredi S Henke L Hidding M Kärgel HJ Lessig R Nagy M Pascali VL Parson W Rolf B Schmitt C Szibor R Teifel-Greding J Krawczak M A new method for the evaluation of matches in non-recombining genomes: application to Y-chromosomal short tandem repeat (STR) haplotypes in European males Forensic Sci Int 2000 114 31 43 10.1016/S0379-0738(00)00287-5 10924848

[B32] Andersen MM Caliebe A Jochens A Willuweit S Krawczak M Estimating trace-suspect match probabilities for singleton Y-STR haplotypes using coalescent theory Forensic Sci Int Genet 2013 7 264 271 10.1016/j.fsigen.2012.11.004 23270696

[B33] Willuweit S Roewer L International Forensic Y Chromosome User Group Y chromosome haplotype reference database (YHRD): update Forensic Sci Int Genet 2007 1 83 87 10.1016/j.fsigen.2007.01.017 19083734

[B34] Parson W Dür A EMPOP - a forensic mtDNA database Forensic Sci Int Genet 2007 1 88 92 10.1016/j.fsigen.2007.01.018 19083735

[B35] Roewer L Croucher PJ Willuweit S Lu TT Kayser M Lessig R de Knijff P Jobling MA Tyler-Smith C Krawczak M Signature of recent historical events in the European Y-chromosomal STR haplotype distribution Hum Genet 2005 116 279 291 10.1007/s00439-004-1201-z 15660227

[B36] Roewer L Male DNA Fingerprints say more Profiles in DNA 2004 7 14 15

[B37] Phillips C Fondevila M Lareu MV A 34-plex autosomal SNP single base extension assay for ancestry investigations Methods Mol Biol 2012 830 109 126 10.1007/978-1-61779-461-2_8 22139656

[B38] Pereira R Phillips C Pinto N Santos C dos Santos SE Amorim A Carracedo A Gusmão L Straightforward inference of ancestry and admixture proportions through ancestry-informative insertion deletion multiplexing PLoS One 2012 7 e29684 10.1371/journal.pone.0029684 22272242 PMC3260179

[B39] Pakstis AJ Fang R Furtado MR Kidd JR Kidd KK Mini-haplotypes as lineage informative SNPs and ancestry inference SNPs Eur J Hum Genet 2012 20 1148 1154 10.1038/ejhg.2012.69 22535184 PMC3476707

[B40] Maguire CN McCallum LA Storey C Whitaker JP Familial searching: A specialist forensic DNA profiling service utilising the National DNA Database® to identify unknown offenders via their relatives - The UK experience Forensic Sci Int Genet 2013 8 1 9 24315582 10.1016/j.fsigen.2013.07.004

[B41] Jeffreys A Genetic Fingerprinting Nat Med 2005 11 1035 1039 10.1038/nm1005-1035 16211029

[B42] Machado H Silva S Would you accept having your DNA profile inserted in the National Forensic DNA database? Why? Results of a questionnaire applied in Portugal Forensic Sci Int Genet 2013 Epub ahead of print 10.1016/j.fsigen.2013.08.014 24315600

[B43] Parson W Strobl C Strobl C Huber G Zimmermann B Gomes SM Souto L Fendt L Delport R Langit R Wootton S Lagacé R Irwin J Evaluation of next generation mtGenome sequencing using the Ion Torrent Personal Genome Machine (PGM) Forensic Sci Int Genet 2013 7 632 639 10.1016/j.fsigen.2013.09.007 24119954

[B44] Budowle B van Daal A Forensically relevant SNP classes Biotechniques 2008 44 603 608 610 18474034 10.2144/000112806

[B45] Allen M Nilsson M Havsjö M Edwinsson L Granemo J Bjerke M Haloplex and MiSeq NGS for simultaneous analysis of 10 STRs, 386 SNPs and the complete mtDNA genome Presentation at the 25th Congress of the International Society for Forensic Genetics 2013 Melbourne 2–7 September 2013

[B46] Bandelt HJ Salas A Current next generation sequencing technology may not meet forensic standards Forensic Sci Int Genet 2012 6 143 145 10.1016/j.fsigen.2011.04.004 21565569

[B47] Tan E Turingan RS Hogan C Vasantgadkar S Palombo L Schumm JW Selden RF Fully integrated, fully automated generation of short tandem repeat profiles Investigative Genet 2013 4 16 10.1186/2041-2223-4-16 PMC3751157 23915594

[B48] Biesecker LG Bailey-Wilson JE Ballantyne J Baum H Bieber FR Brenner C Budowle B Butler JM Carmody G Conneally PM Duceman B Eisenberg A Forman L Kidd KK Leclair B Niezgoda S Parsons TJ Pugh E Shaler R Sherry ST Sozer A Walsh A DNA Identifications after the 9/11 World Trade Center Attack Science 2005 310 1122 1123 10.1126/science.1116608 16293742

[B49] Dolan SM Saraiya DS Donkervoort S Rogel K Lieber C Sozer A The emerging role of genetics professionals in forensic kinship DNA identification after a mass fatality: lessons learned from Hurricane Katrina volunteers Genet Med 2009 11 414 417 10.1097/GIM.0b013e3181a16ccc 19444129

[B50] Huffine E Crews J Kennedy B Bomberger K Zinbo A Mass identification of persons missing from the break-up of the former Yugoslavia: structure, function, and role of the International Commission on Missing Persons Croat Med J 2001 42 271 275 11387637

[B51] Corach D Sala A Penacino G Iannucci N Bernardi P Doretti M Fondebrider L Ginarte A Inchaurregui A Somigliana C Turner S Hagelberg E Additional approaches to DNA typing of skeletal remains: the search for “missing” persons killed during the last dictatorship in Argentina Electrophoresis 1997 18 1608 1612 10.1002/elps.1150180921 9378130

[B52] Levitt M Forensic databases: benefits and ethical and social costs Br Med Bull 2007 83 235 248 10.1093/bmb/ldm026 17906329

